# Mycelium-Based Biocomposites as Sustainable Polymer Alternatives: Engineering Strategies, Structure–Property Relationships and Circular Packaging Applications

**DOI:** 10.3390/polym18161946

**Published:** 2026-08-08

**Authors:** Shuai Yuan, Chen Chen, Wei Xu

**Affiliations:** College of Furnishings and Industrial Design, Nanjing Forestry University, Nanjing 210037, China; ys8241421619@njfu.edu.cn (S.Y.); cchen1990@njfu.edu.cn (C.C.)

**Keywords:** mycelium-based composites, bio-based polymer composites, biodegradable composites, structure–property relationships, sustainable packaging, circular economy

## Abstract

Mycelium-based composites (MBCs) represent a new class of bio-based polymer alternatives, where fungal networks act as natural binders within lignocellulosic composite architectures. This review systematically summarises the development of mycelium-based packaging materials from early laboratory research to industrial applications since the pioneering work of Ecovative Design in 2007. The review analyses how fungal strains and substrate compositions regulate the mechanical properties, density, hydrophobicity, and biodegradability of MBCs. Additionally, reinforcement strategies, including natural fibre reinforcement, nanomaterial modification, densification treatment, and process optimisation, are discussed for their roles in improving material performance. Furthermore, current commercial applications of mycelium-based packaging in electronics, food, cosmetics, and construction are reviewed, together with the major challenges limiting large-scale industrialisation, such as production scalability, cost competitiveness, and batch variability. Future research directions, including strain engineering, intelligent living materials, and automated biomanufacturing, are also outlined. Overall, this review provides a comprehensive reference for the design, optimisation, and commercial development of mycelium-based packaging materials within the framework of a circular bioeconomy.

## 1. Introduction

Mycelium is the vegetative structure of fungi, consisting of an interconnected three-dimensional network of filamentous hyphae [1]. Through controlled cultivation processes, fungal mycelium can colonise and bind lignocellulosic agricultural residues, such as straw, sawdust, and rice husks, into lightweight materials with predefined geometries and functional properties [2].

Mycelium-based materials broadly refer to fungal-derived biomaterials fabricated through mycelial growth, including pure mycelium films, leather-like materials, foams, and mycelium-based composites (MBCs). Pure mycelium materials are composed predominantly of fungal biomass and are commonly fabricated as films, membranes, or leather-like structures, whereas MBCs are formed by integrating fungal mycelium with lignocellulosic particulate or fibrous substrates. Among these categories, MBCs have attracted particular attention in sustainable packaging applications owing to their low density, potential for biological degradation, and tunable mechanical properties. In this review, “fungal-derived materials” is used as the general term for the broader material family, while “MBCs” specifically refers to mycelium-bound lignocellulosic composites.

For decades, the packaging industry has relied heavily on expanded polystyrene (EPS) because of its excellent cushioning performance, low cost, and lightweight characteristics [3]. However, the extensive use of EPS has generated substantial environmental concerns. Owing to its petrochemical origin, environmental persistence, and limited biodegradability, discarded EPS contributes to long-term plastic accumulation, fragmentation, and associated ecological burdens [4,5,6,7]. Moreover, EPS recycling remains economically challenging and can involve considerable collection, transportation, and processing requirements, limiting its compatibility with emerging circular economy strategies. These concerns, together with regulatory restrictions on single-use plastics, corporate carbon-neutrality commitments, and increasing demand for renewable packaging materials, have accelerated the search for biologically derived alternatives [8,9,10].

Against this background, mycelium-based packaging materials have emerged as promising bio-based alternatives to conventional polymer foams. Unlike synthetic foams manufactured through petrochemical conversion and physical or chemical foaming, MBCs are formed through biological self-assembly driven by fungal growth. By utilising agricultural by-products as lignocellulosic substrates, these materials can reduce dependence on virgin synthetic feedstocks and provide potential pathways for biological degradation at the end of life [11]. However, their environmental advantages are not automatic and depend on feedstock transportation, substrate sterilisation, cultivation-related environmental control, drying, thermal inactivation, surface treatment, and the end-of-life scenario included within the assessment boundary [12,13]. This “growing rather than manufacturing” paradigm therefore represents a potential transition from conventional extractive production towards biofabrication-based circular material systems, provided that energy-intensive processing steps and disposal conditions are appropriately managed.

Recent research has shifted from basic fabrication feasibility towards application-oriented material engineering and manufacturing control. Studies have expanded fungal biodiversity and agricultural-residue screening for packaging biofoams, showing that fungal species, substrate composition, moisture content, cultivation conditions, and post-processing jointly regulate density, water response, and mechanical performance [14,15,16]. In parallel, natural-fibre reinforcement, nanostructured interfaces, and hybrid material architectures have been investigated to improve strength, durability, and multifunctionality [17,18,19]. Data-driven approaches have also been introduced to predict the mechanical properties of MBCs from interacting biological, compositional, and physical variables [20]. At the manufacturing level, increasing attention is being directed towards process standardisation, quality control, automation, and the barriers preventing laboratory-scale prototypes from becoming consistent industrial products [9,21].

Despite these advances, several limitations continue to restrict a comprehensive assessment of MBCs for packaging applications. Reported property ranges are frequently compared across studies without sufficient consideration of differences in fungal strain, substrate chemistry, density, cultivation conditions, post-processing, specimen geometry, and testing protocol. Reinforcement strategies are generally evaluated according to performance gains, while their additional cost, processing complexity, influence on biodegradation, and suitability for industrial scale-up receive less systematic attention. Pure mycelium materials and lignocellulosic MBCs are also sometimes discussed as a single material category despite their distinct production routes, structures, costs, and application profiles. In addition, commercialisation assessments remain limited by incomplete cost data, batch-to-batch variability, inconsistent life cycle boundaries, insufficiently harmonised quality standards, and unresolved biosafety considerations [9,16,21].

**Figure 1:**
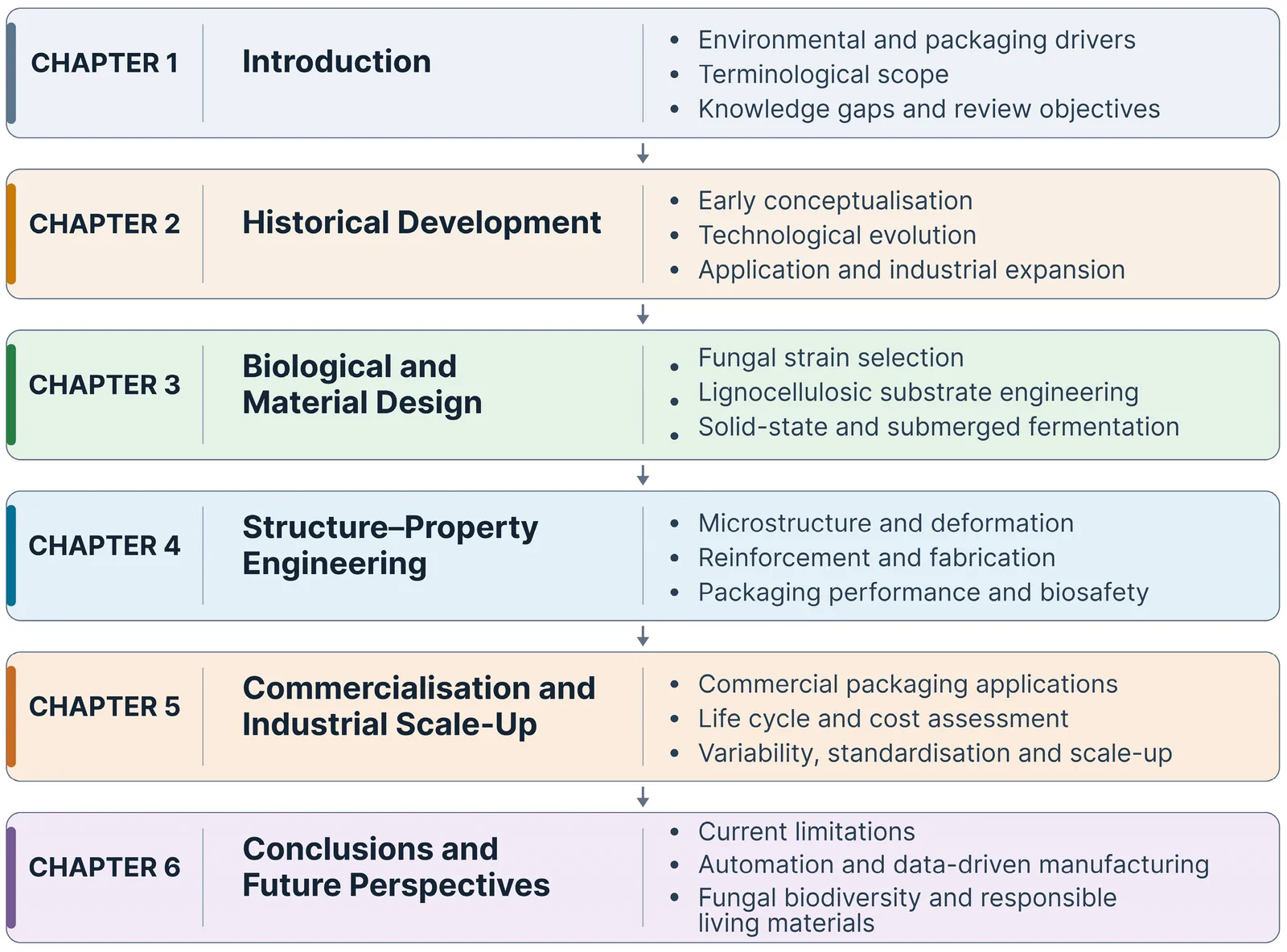
Chapter-aligned framework linking the historical development, biological and material design, structure–property engineering, commercialisation, industrial scale-up and future research priorities of mycelium-based packaging materials.

Accordingly, this review aims to establish an integrated framework linking biological selection, material architecture, fabrication conditions, engineering performance, packaging requirements, and industrial implementation. It first examines the historical development of fungal-derived materials and the roles of fungal strains, lignocellulosic substrates, and cultivation routes in regulating MBC structure and performance. Reinforcement and optimisation strategies—including natural-fibre incorporation, nanomaterial modification, densification, surface treatment, and process engineering—are then evaluated in relation to performance improvement, production complexity, cost, durability, and end-of-life implications. Commercial packaging applications are assessed together with life cycle evidence, batch variability, quality control, biosafety, and scale-up barriers. Finally, research and industrialisation priorities are identified from the perspectives of fungal biodiversity, strain engineering, automated biomanufacturing, standardised testing, data-driven prediction, and responsibly designed living materials. Through this structure, the review connects material-level structure–property relationships with the functional and manufacturing requirements governing the practical adoption of mycelium-based packaging, as illustrated in Figure 1.

## 2. Historical Development of Mycelium-Based Materials

### 2.1. Early Conceptualisation and Exploratory Research

Systematic research into mycelium-based materials as sustainable alternatives to petroleum-derived materials began in the early 2000s. Inspired by the natural ability of fungal mycelium to bind and consolidate lignocellulosic particles, Eben Bayer and Gavin McIntyre founded Ecovative Design in 2007 while studying at Rensselaer Polytechnic Institute [2,22]. Their pioneering work demonstrated that fungal mycelium could function as a biological adhesive capable of transforming loose agricultural waste into lightweight structural materials with customised geometries.

This early stage established the biological and physicochemical foundations of MBCs. Studies revealed that chitin within fungal cell walls contributes significantly to the mechanical rigidity of mycelial networks, while hydrophobin proteins provide intrinsic hydrophobic characteristics [23,24]. Saprophytic fungi, particularly white-rot fungi, were shown to secrete extracellular enzymes such as lignin peroxidase, manganese peroxidase, and laccase, enabling the efficient degradation and reorganisation of lignocellulosic substrates into interconnected biodegradable structures [25,26].

### 2.2. Technological Development and Expansion of Academic Research

A major milestone in the commercialisation of mycelium-based materials was achieved when Ecovative Design introduced the world’s first commercial mycelium packaging products in 2007 [27]. Researchers subsequently demonstrated that a wide range of agricultural by-products, including hemp hurds, sawdust, cottonseed hulls, and oat husks, could serve as effective substrates for fungal growth [28]. By inoculating these substrates into moulds under controlled environmental conditions, foam-like composite structures could be produced within approximately one week through near-net-shape biofabrication [25].

Following these industrial developments, academic interest in MBCs increased rapidly. From 2010 onwards, systematic investigations evaluated the thermal, mechanical, and physical properties of MBCs in comparison with EPS and other conventional foams [29,30,31,32,33]. Representative comparisons between MBCs and EPS are summarised in Table 1. Studies demonstrated that although untreated MBCs generally exhibited lower compressive strength than commercial EPS, densification treatments and hot pressing markedly improved their stiffness and structural integrity [34,35].

**Figure 2:**
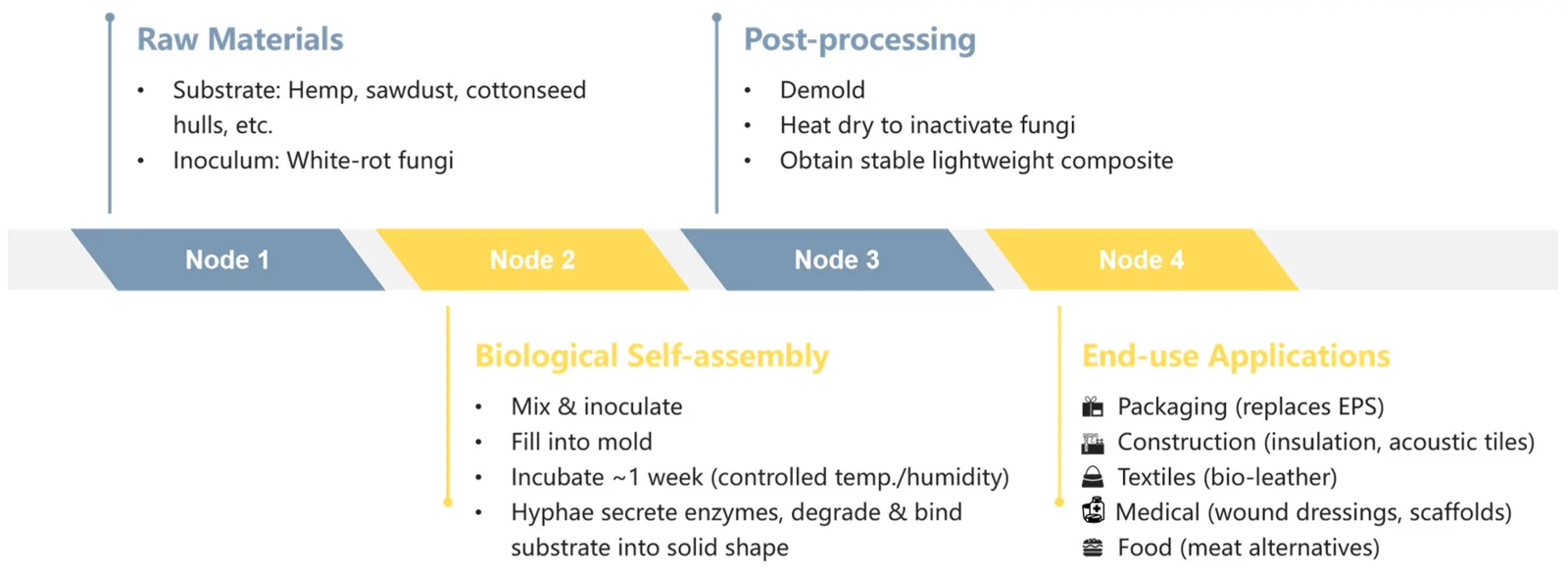
Standard production workflow of MBCs, from lignocellulosic feedstock preparation to final products.

During this phase, the research focus gradually shifted from basic material feasibility towards performance optimisation and application-oriented engineering. Increasing attention was devoted to enhancing interfacial bonding between fungal mycelium and lignocellulosic substrates through substrate pretreatment, density control, and process optimisation [40]. Simultaneously, the standardised production workflow of MBCs—including substrate preparation, sterilisation, inoculation, incubation, moulding, and thermal inactivation—became progressively established. The typical manufacturing process of mycelium-based composites is illustrated in Figure 2.

These technological advances enabled mycelium-based materials to transition from experimental biomaterials into viable candidates for thermal insulation, cushioning, and protective packaging applications [27]. Moreover, collaborations with multinational companies such as Dell signalled the growing industrial interest in sustainable fungal-derived packaging systems [2].

### 2.3. Diversification of Applications and Industrial Expansion

Over the past two decades, the application scope of mycelium-based materials has expanded substantially beyond protective packaging. Owing to their biodegradability, low density, biocompatibility, and structural versatility, MBCs and related fungal-derived materials are increasingly being explored for use in construction panels, acoustic insulation, textiles, leather-like materials, biomedical scaffolds, and wearable bioelectronics [8,41,42,43].

This diversification reflects a broader transition in the perception of fungal biomaterials: from low-cost biodegradable substitutes towards high-value functional materials with programmable properties. In particular, advances in pure mycelium cultivation through submerged fermentation (SmF) have enabled the development of flexible mycelium membranes and leather-like materials with enhanced uniformity and mechanical performance [11,43].

From an industrial perspective, commercialisation remains highly concentrated among a small number of pioneering enterprises. Patent analyses indicate that Ecovative Design controls approximately 45% of global patents related to mycelium materials, particularly in packaging and structural applications, while MycoWorks focuses primarily on high-end fungal leather technologies [44]. This concentration highlights both the technological maturity achieved by early innovators and the substantial barriers that still exist for large-scale industrial entry.

Simultaneously, growing global interest in circular bioeconomy strategies, carbon neutrality targets, and sustainable manufacturing has accelerated investment in fungal biomaterials [45,46,47]. Therefore, mycelium-based materials have progressively evolved from niche experimental products into an emerging platform technology for sustainable material systems.

**Figure 3:**
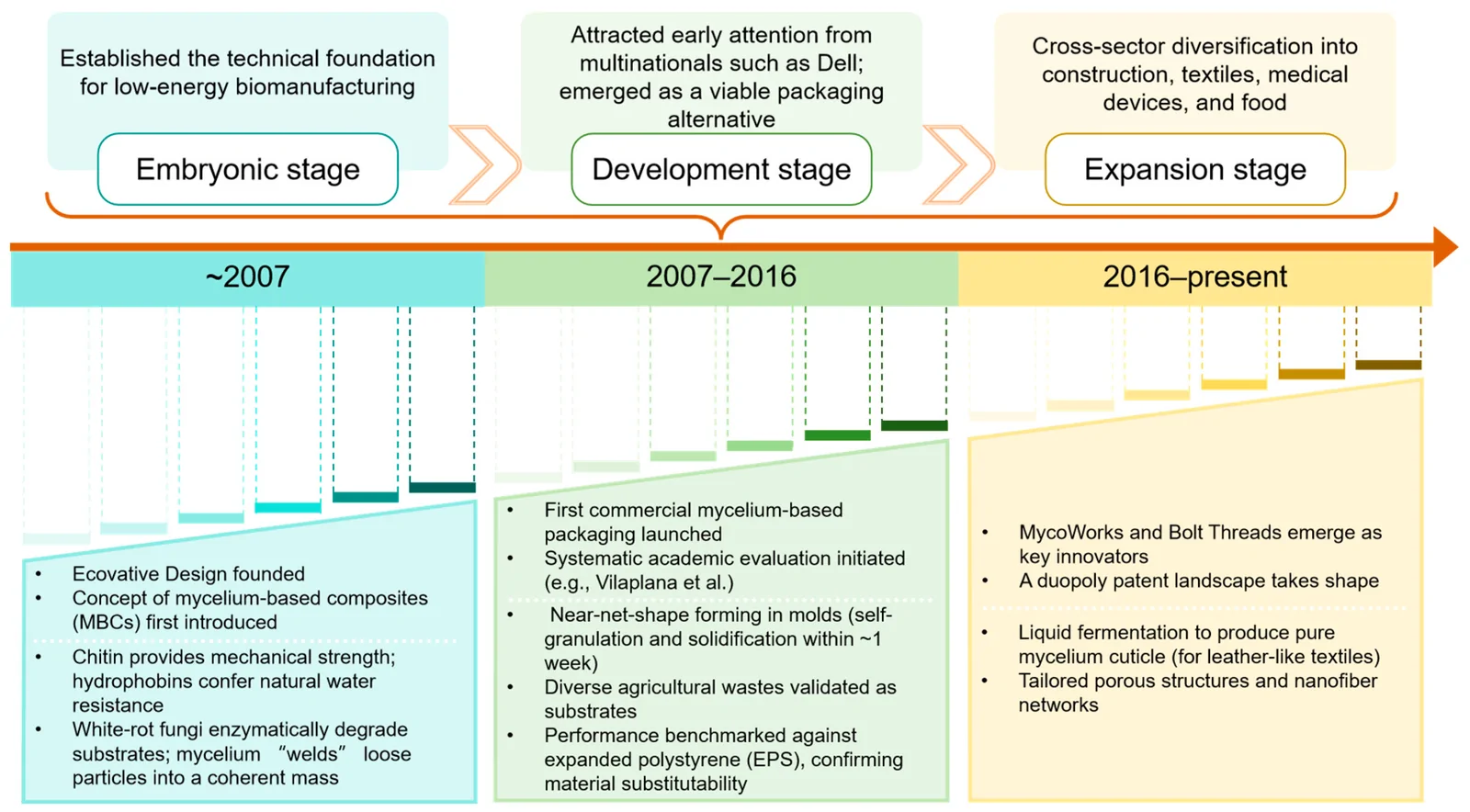
Evolutionary trajectory of mycelium-based materials from laboratory-scale biofabrication to industrial and commercial applications [29].

The historical evolution of mycelium-based materials from early laboratory exploration to industrial implementation is summarised in Figure 3.

## 3. Biological and Material Design of Mycelium-Based Materials

### 3.1. Fungal Strain Selection and Regulation of Material Properties

#### 3.1.1. Dominant Fungal Species Used in MBCs

Current research on MBCs primarily focuses on basidiomycete fungi, particularly *Ganoderma lucidum*, *Pleurotus ostreatus*, and *Trametes versicolor* [48,49,50]. These fungal species exhibit strong lignocellulose-degrading capabilities and stable mycelial growth behaviour, making them highly suitable for composite fabrication.

Differences in hyphal morphology, cell wall composition, growth kinetics, and extracellular enzyme secretion can contribute to variations in MBC performance [51]. However, reported differences among fungal species should not be interpreted as intrinsic or universal property rankings. Under specific combinations of substrate, cultivation and post-processing conditions, *G. lucidum* has been associated with relatively stiff composite structures, whereas *P. ostreatus* may generate more compliant and flexible networks, and *T. versicolor* often exhibits intermediate responses [11,25]. The upper and lower property values reported for each species also vary substantially with fungal strain or isolate, substrate chemistry, particle size, moisture content, cultivation time, material density, pressing conditions, specimen geometry, and testing protocol [40,48,51].

Representative reported ranges and qualitative tendencies in the mechanical and cell-wall characteristics of commonly used fungal species are summarised in Table 2.

#### 3.1.2. Mechanisms Governing Strain-Dependent Material Performance

Fungal identity influences MBC performance through differences in hyphal morphology, cell-wall composition, growth kinetics, and extracellular enzyme secretion. Chitin and β-glucans contribute to the rigidity and tensile behaviour of individual hyphae; however, the bulk density and mechanical performance of MBCs arise from the coupled effects of the fungal network, lignocellulosic substrate skeleton, interfacial bonding, and processing history [11,23,25,40]. Cell-wall composition should therefore be considered one biological design variable rather than a direct predictor of composite strength.

Extracellular oxidative enzymes—including laccases and manganese peroxidases—strongly influence substrate degradation kinetics and interfacial bonding behaviour [27,52]. During fungal colonisation, these enzymes selectively decompose lignin and reorganise lignocellulosic structures, thereby promoting mechanical interlocking between the mycelium and substrate particles [25]. This process has increasingly been interpreted as a form of enzymatic interfacial engineering, in which fungal metabolism actively regulates the microscopic architecture and macroscopic mechanical performance of the composite material.

Ligninolytic activity can promote substrate colonisation and interfacial contact by partially modifying lignocellulosic surfaces. However, excessive degradation may weaken the load-bearing substrate skeleton and reduce structural integrity. Fungal strain selection therefore requires a balance between rapid colonisation, effective interfacial binding, and preservation of substrate strength rather than simply maximising lignin degradation [27,52,53].

#### 3.1.3. Emerging Fungal Species and Specialised Cultivation Systems

Research on fungal-derived materials is also expanding beyond conventional lignocellulosic MBCs towards pure mycelium films, membranes, fibres, and leather-like sheets. These materials are produced by cultivating fungal biomass separately from a particulate reinforcing substrate, including through SmF or related liquid-culture routes, which can provide greater control over biomass morphology, purity, and structural uniformity [11,51,54]. However, biomass recovery, sheet formation, drying, and subsequent post-processing introduce production requirements that differ substantially from those of mould-grown MBCs. Pure mycelium materials should therefore be evaluated separately from lignocellulosic composites in terms of performance, cost, and industrial scale-up.

These developments demonstrate that fungal selection cannot be treated as an isolated design variable, because its effects emerge through interactions with cultivation route, substrate chemistry, and subsequent processing conditions.

### 3.2. Selection and Engineering of Lignocellulosic Substrates

#### 3.2.1. Agricultural Residues as Primary Substrate Sources

The fabrication of MBCs relies heavily on lignocellulosic agricultural residues, which serve both as nutrient sources and structural frameworks for fungal growth [55]. Typical substrates include sawdust, hemp hurds, wheat straw, rice husks, oat hulls, bagasse, and cottonseed hulls [56,57,58]. These materials are primarily composed of cellulose, hemicellulose, and lignin, the relative proportions of which strongly influence fungal colonisation behaviour and composite performance.

Regional substrate availability and substrate–fungus compatibility further influence material design. Studies involving California walnut shells, cottonseed hulls, and other agricultural by-products have demonstrated that substrate–fungus compatibility significantly influences composite morphology and porosity [2,56]. Certain fungal species, such as *Volvariella volvacea*, exhibit unique growth characteristics capable of generating composites with highly differentiated pore structures under specific substrate compositions [59].

Substrates with higher lignin content generally contribute to improved hydrophobicity, while more recalcitrant substrates can stimulate increased chitin synthesis and denser mycelial network formation [1,25]. As a result, substrate chemistry directly affects the balance between biodegradability, mechanical strength, and environmental resistance.

#### 3.2.2. Influence of Substrate Structure on Composite Performance

Among substrate parameters, particle size distribution plays a particularly important role in determining the density, porosity, and mechanical behaviour of MBCs [60]. Smaller particles provide larger specific surface areas, facilitating tighter mycelial entanglement and enhanced interfacial bonding. As a result, composites fabricated from fine sawdust particles often exhibit higher density and compressive strength [58].

However, excessively fine particles may inhibit oxygen diffusion and fungal metabolism, negatively affecting mycelial growth and structural integrity. In contrast, mixed coarse–fine particle systems can generate hierarchical pore architectures that simultaneously optimise strength and lightweight performance [61].

Representative studies examining the effects of substrate selection, particle architecture, inoculum level, and moisture-related preparation parameters on MBC structure and mechanical performance are summarised in Table 3.

These findings demonstrate that substrate engineering is a multi-scale optimisation process involving trade-offs among nutrient availability, oxygen and moisture transport, fungal colonisation, porosity, density, and interfacial load transfer. No universally optimal particle size or substrate composition can therefore be defined independently of fungal strain, cultivation conditions, target density, and packaging-performance requirements.

### 3.3. Solid-State and Submerged Fermentation Systems

Solid-state fermentation (SSF) currently represents the dominant manufacturing approach for mycelium-based packaging materials because it enables the direct utilisation of agricultural waste under relatively low-cost and low-energy conditions [55]. In SSF systems, fungal mycelium colonises solid substrates within moulds, allowing simultaneous growth and shaping of composite structures.

In contrast, SmF is primarily employed for the production of pure mycelium films, membranes, and leather-like materials requiring higher structural uniformity and surface quality [54]. Although SmF offers superior control over mycelial morphology and purity, its higher operational complexity and production costs currently limit its application in large-scale packaging production.

Beyond substrate selection, the performance of mycelium-based biofoams is strongly governed by cultivation-related growth factors, including incubation temperature, moisture content, and spawn loading. Nashiruddin et al. demonstrated that these parameters substantially affected mycelial growth, dry density, and compressive strength during the production of *P. ostreatus*-based biofoam. In their study, the highest dry density was obtained at 50% moisture content, whereas the optimum compressive strength was achieved at 40% spawn loading. These findings confirm that fermentation conditions should be regarded as active design variables rather than merely operational settings when tailoring mycelium-based packaging materials [64].

A technical comparison between SSF and SmF systems is presented in Table 4.

Together, fungal strain selection, substrate characteristics and cultivation route determine the biological and structural basis for subsequent property regulation, reinforcement and packaging-oriented engineering of MBCs.

## 4. Structure–Property Engineering and Packaging Performance

MBCs are biofabricated materials in which fungal mycelium functions as a biological binder that interconnects lignocellulosic particles into integrated three-dimensional structures [67]. Compared with pure mycelium materials, composite systems significantly improve the structural integrity, mechanical performance, and functional tunability of fungal biomaterials [40,68,69]. Therefore, the development of reinforcement strategies and process optimisation approaches has become a central research direction in advancing MBCs towards practical engineering and packaging applications.

### 4.1. Structural Characteristics of Mycelium-Based Composites

The microstructure of MBCs is composed primarily of fungal mycelial networks, lignocellulosic substrate particles, interfacial bonding regions, and hierarchical pore systems [67,70]. During fungal colonisation, mycelial hyphae penetrate and entangle substrate particles, forming interconnected fibrous networks that act as natural reinforcement and binding phases.

The porous architecture of MBCs plays a dual role in determining material performance. On one hand, interconnected pore networks reduce density and enhance thermal insulation and acoustic absorption properties [71,72,73,74]. On the other hand, excessive porosity may compromise mechanical strength and structural stability. Therefore, the balance between porosity and densification is a key challenge in the engineering design of MBCs.

Interfacial bonding between fungal hyphae and substrate particles is particularly important for mechanical integrity. The combination of biochemical adhesion and physical interlocking mechanisms determines fracture resistance, toughness, and fatigue behaviour [75,76]. Representative structural elements and their corresponding effects on material performance are summarised in Table 5.

### 4.2. Mechanical Behaviour, Deformation Mechanisms and Property Variability

The mechanical behaviour of MBCs is strongly influenced by their porous hierarchical structure and viscoelastic fungal networks. Under compressive loading, MBCs typically exhibit a three-stage stress–strain response consisting of: (i) an initial elastic deformation region, (ii) a plateau region associated with progressive pore collapse and energy absorption, and (iii) a densification stage characterised by rapid stress increase following pore closure [35,60].

This deformation behaviour is strongly dependent on the internal density, pore architecture and interfacial bonding of the composite. Reported elastic modulus values of approximately 35–97 MPa should therefore not be interpreted as an intrinsic property range for all MBCs [40,48]. The lower and upper bounds vary with fungal species, substrate composition and particle size, mycelial growth extent, moisture content, specimen density, fibre orientation, pressing conditions, specimen geometry and mechanical testing protocol [30,40,56,58,60]. Comparisons between studies should consequently be made only when density, conditioning conditions and test methods are reported consistently.

For protective packaging, elastic modulus and compressive strength alone are insufficient to establish functional equivalence with petroleum-derived foams. Relevant performance indicators also include plateau stress, energy absorption per unit mass, recovery after repeated compression, creep under sustained loading, puncture resistance, vibration damping and mechanical retention after humidity conditioning [35,46,60,77]. Reporting these parameters together would provide a more application-oriented evaluation of MBCs.

Densification generally increases strength, stiffness and dimensional stability by reducing macroporosity and improving contact between substrate particles [40,51,61,78]. However, maximum densification is not necessarily optimal for packaging. Higher density increases material consumption and transportation mass, while severe pressing may reduce thermal insulation, cushioning efficiency and biodegradation rate [40,78,79]. Processing should therefore be optimised for specific energy absorption and recovery rather than for maximum compressive strength alone.

**Figure 4:**
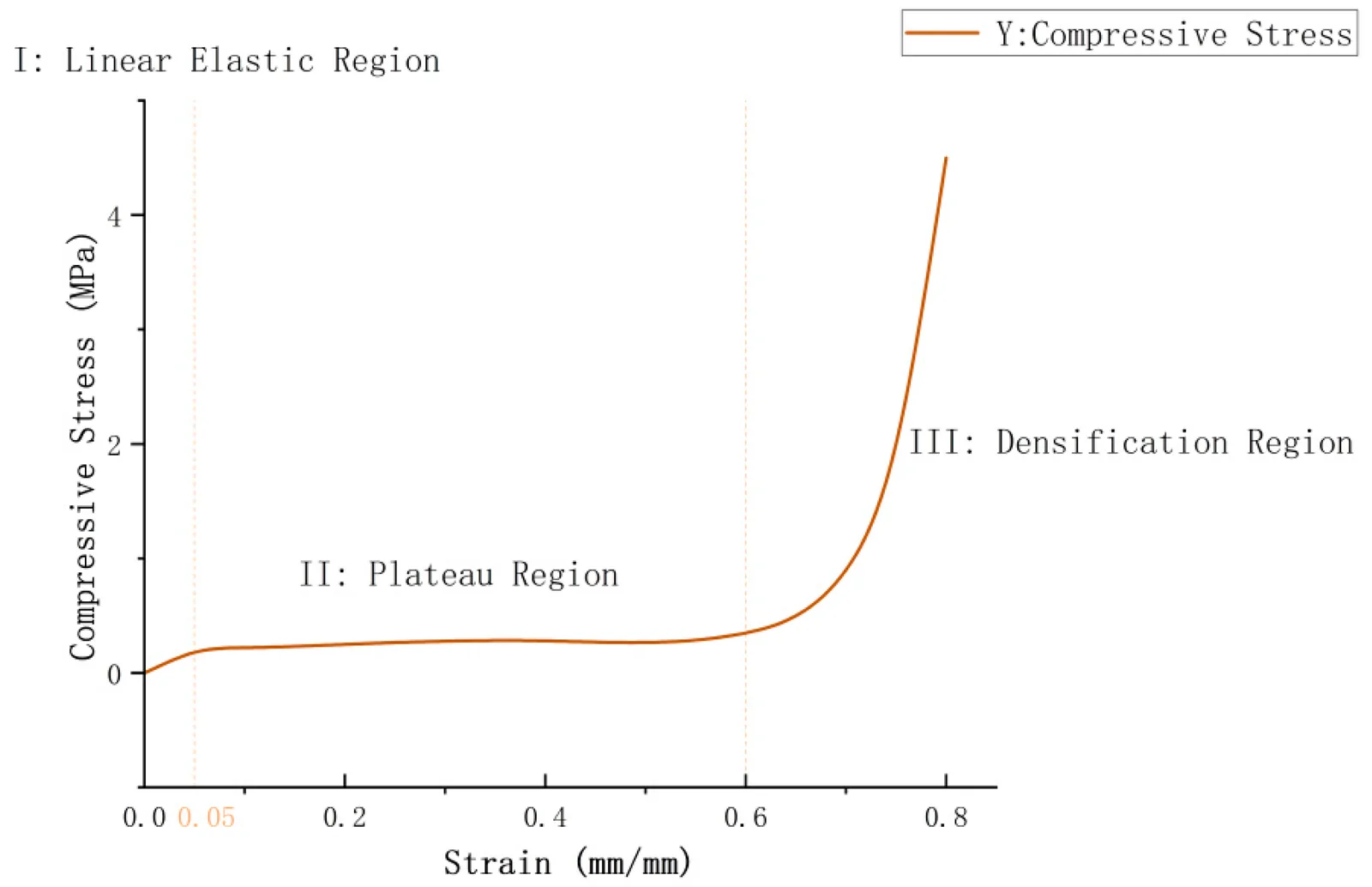
Schematic compressive stress–strain response and deformation mechanisms of mycelium-based composites.

The representative compressive stress–strain response of MBCs and the corresponding deformation stages are illustrated schematically in Figure 4.

### 4.3. Reinforcement Strategies and Comparative Trade-Offs

To further improve the structural performance of MBCs, various reinforcement strategies have been developed, including natural fibre reinforcement, nanomaterial incorporation, and hybrid composite engineering.

Natural fibres such as flax, hemp, jute and bamboo can provide continuous or semi-continuous load-transfer pathways within the otherwise particulate substrate network. Their effectiveness depends on fibre aspect ratio, orientation, spatial distribution and adhesion to the surrounding mycelial matrix [17,80,81]. However, natural fibres are intrinsically hydrophilic and may absorb moisture, swell or act as preferential water-transport pathways. Fibre agglomeration and poor dispersion can also produce local defects rather than reinforcement [17,80]. During cultivation, some lignocellulosic fibres may be partially degraded by fungal enzymes, while excessive fibre contents may restrict oxygen transfer and non-uniformly alter mycelial colonisation [17,76,80]. Fibre treatment and controlled alignment can mitigate these limitations, but they add processing steps, energy demand and cost.

Carbon nanotubes can improve electrical conductivity and, under appropriate conditions, reinforce the mycelial network. In living composite systems, conductive networks may additionally support sensing or self-regenerative functions [18]. However, these advantages depend on uniform nanotube dispersion and continued biological activity. After complete thermal inactivation, claims of biologically mediated self-repair should no longer be applied [18,82]. Nanotube cost, dispersion difficulty, occupational exposure and end-of-life ecotoxicity also limit their immediate suitability for high-volume disposable packaging [18,68].

Cellulose nanocrystals and chitin nanofibres may provide renewable routes for nanoscale reinforcement and improved interfacial stress transfer [68]. However, their hydrophilicity can increase moisture sensitivity, while their isolation, purification and dispersion may require substantial energy and processing resources. Their effectiveness and life-cycle impacts in MBC packaging systems therefore remain to be systematically evaluated [68].

The available studies do not yet support a statistically valid pooled estimate of reinforcement gains because fungal species, substrate composition, additive loading, density and testing protocols differ substantially. A qualitative comparison nevertheless indicates distinct trade-offs. Densification is currently the simplest and most scalable strategy, but it increases mass and may reduce cushioning and insulation [40,78,79]. Natural-fibre reinforcement offers relatively low material cost and compatibility with existing lignocellulosic feedstocks, although moisture sensitivity and fibre dispersion remain important limitations [17,80]. Nanoscale reinforcements can provide larger functional gains, including electrical conductivity, but introduce higher cost, dispersion complexity and end-of-life uncertainty [18,68]. Hybrid polymer systems can improve water resistance and mechanical stability, but their biodegradability depends on the identity, loading and continuity of the added polymer phase. For disposable packaging, moderate densification combined with locally sourced natural fibres currently appears to provide the most practical balance between performance, cost and manufacturing complexity [17,18,40,78,79,80].

A qualitative comparison of representative reinforcement and surface-modification strategies is provided in Table 6. Because the available studies differ substantially in fungal strain, substrate, additive loading, final density, specimen geometry and testing protocol, pooled percentage improvements and universal performance rankings are not assigned.

### 4.4. Fabrication Processes, Surface Treatments and Durability

Fabrication parameters exert a decisive influence on the morphology and performance of MBCs. Key processing variables include substrate particle size, cultivation humidity, incubation time, temperature, compaction pressure, and post-treatment conditions [40,55,78,79].

During cultivation, fungal growth kinetics directly affect network density and substrate degradation behaviour. Shorter cultivation periods generally preserve substrate integrity and yield denser composites, whereas excessive incubation may result in over-degradation, increased porosity, and reduced mechanical stability [55,76,79].

Post-growth drying and hot pressing are commonly used to terminate fungal activity, reduce moisture content and consolidate the composite structure. Increasing pressing temperature, pressure or duration generally improves dimensional stability and interparticle contact, but excessive treatment can embrittle the material, collapse the cushioning pore structure and increase production energy demand [40,51,78,79,82]. Processing conditions should therefore be selected according to the required balance among mechanical strength, recovery, insulation and manufacturing efficiency.

Surface coatings can reduce water absorption and delay moisture-induced loss of mechanical performance [36]. For example, beeswax- and coconut-oil-based treatments have been reported to improve the water barrier behaviour of MBCs; a formulation containing a high proportion of beeswax reduced water absorption and delayed visible fungal colonisation under the investigated conditions [36]. These results demonstrate improved resistance to wetting and biological colonisation during use, but they do not by themselves demonstrate complete biodegradation or mineralisation after disposal.

Coating selection creates an inherent trade-off between service durability and end-of-life biodegradation. Hydrophobic layers that effectively prevent water and microbial penetration during use may also delay fragmentation and composting after disposal [36,39,83]. Petroleum-derived or non-biodegradable polymer coatings can further introduce persistent residues, whereas a coating described as natural cannot automatically be assumed to produce no harmful degradation products. Coated MBCs should therefore be evaluated using coating-specific disintegration, aerobic biodegradation or mineralisation, leachate ecotoxicity and residual-particle testing [21,36,39]. For food-contact packaging, chemical migration and sensory effects should also be assessed [10,84]. Thin, localised and renewable coatings, or barrier layers designed to detach or become permeable under composting conditions, may provide a more favourable balance between shelf-life protection and biological end-of-life treatment.

Additionally, near-net-shape biofabrication enables the direct growth of MBCs into customised geometries without extensive machining or secondary processing [66]. This manufacturing strategy significantly reduces material waste and production complexity, representing a major advantage over conventional petroleum-based foams.

### 4.5. Packaging Performance and Biosafety Requirements

The suitability of MBCs for packaging depends on the requirements of the intended product rather than on a single material property. Low-density MBCs are primarily relevant to cushioning, void filling and thermal insulation, whereas densified materials may be more appropriate for trays, inserts and semi-rigid protective components [15,46,77,85]. Application-specific evaluation should include density, compressive energy absorption, recovery after repeated loading, vibration damping, puncture resistance, thermal conductivity, water uptake and performance after humidity exposure [15,46,77,85].

MBCs should also be considered within the broader development of bio-based foams. Guar-gum/cellulose foams have, for example, been evaluated using density, thermal conductivity and mechanical response as combined insulation criteria [86]. Although their formulation and manufacturing route differ substantially from those of MBCs, this work illustrates the importance of benchmarking thermal and mechanical performance simultaneously when assessing renewable foam alternatives.

Selected densified MBC formulations have approached the compressive or flexural performance of particular EPS products under specific test conditions [15,46,77,85,87]. However, such comparisons must be interpreted cautiously because EPS grade, density, specimen geometry, conditioning and test protocol vary between studies [15,40,46,77]. MBCs generally exhibit higher density, greater moisture sensitivity and more variable recovery behaviour than conventional packaging foams. Likewise, reported mass loss or physical disintegration over several weeks or months should not automatically be described as complete biodegradation [36,37,38,39]. End-of-life behaviour depends on substrate composition, density, thermal treatment, coating formulation, microbial activity, temperature and moisture, and should be distinguished among home composting, industrial composting, soil burial and uncontrolled environmental exposure [36,37,38,39].

Thermal inactivation at approximately 100–120 °C can substantially reduce fungal viability and prevent continued growth or sporulation, provided that the treatment reaches all regions of the product [82]. However, loss of viability alone does not demonstrate the absence of pre-formed mycotoxins, allergenic proteins, volatile metabolites or contaminants introduced through the substrate and cultivation environment [84,88]. Biosafety assessment should therefore begin with well-characterised, non-pathogenic and preferably biosafety-level-1 strains that are not known to produce relevant mycotoxins. Production controls should include strain identity verification, substrate contaminant screening, post-treatment viability or colony-forming-unit testing, and monitoring of unwanted moulds and bacteria [82,84,88].

For packaging applications, additional testing should consider fungal spores and fragments, relevant secondary metabolites, allergenic or irritant components, volatile organic compounds and microbial regrowth under high-humidity conditions [84]. Where direct food contact is intended, chemical and biological migration testing should be conducted under the applicable contact conditions [10]. Thermal inactivation should therefore be treated as one component of a broader risk-control system rather than as sufficient evidence of consumer safety [82,84,88].

## 5. Commercialisation, Sustainability and Industrial Scale-Up

MBCs are increasingly recognised as emerging sustainable alternatives to petroleum-derived packaging materials, particularly EPS and polyurethane foams [8,46]. Driven by global carbon-reduction strategies, circular bioeconomy initiatives and growing environmental awareness, MBCs have progressed from laboratory-scale prototypes towards early commercial implementation, although reproducible high-throughput manufacturing remains constrained by long cultivation cycles, process variability and the need for controlled production environments [14,68,89].

This chapter critically evaluates the current market applications, industrial development trends, commercialisation barriers, and future opportunities associated with mycelium-based packaging materials.

### 5.1. Industrial Development and Life Cycle Assessment

Available life cycle assessments generally indicate lower climate-change and fossil-energy burdens for the MBC systems examined; however, the results are not directly interchangeable because the studies differ in functional units, material density, electricity mix, system boundaries and end-of-life assumptions [12,13,90]. Consequently, the environmental advantages of MBCs should be interpreted as system- and scenario-dependent rather than as an inherent property of all mycelium-based products.

Using the protective packaging required for one 32-inch television as the functional unit, Zoungrana et al. reported a global warming potential of 1.32 kg CO_2_ eq for the lowest-density mycelium-based foam, compared with 3.35 kg CO_2_ eq for expanded polystyrene. However, the impacts of the higher-density mycelium foams increased to 2.16 and 3.24 kg CO_2_ eq, respectively, showing that the environmental advantage diminished as material density and the mass required to provide the same packaging volume increased [12]. Density and functional performance must therefore be considered when comparing packaging systems, rather than relying exclusively on impacts expressed per kilogram of material.

Although the assessment by Zoungrana et al. covered raw-material acquisition, manufacturing, transportation and end-of-life treatment, infrastructure, machinery and maintenance were excluded. In addition, manufacturing energy was represented by an aggregate input of approximately 4–6 MJ kg^−1^ rather than by direct measurements of individual operations such as substrate sterilisation or pasteurisation, controlled incubation, climate regulation and final drying [12]. Enarevba and Haapala explicitly represented pasteurisation, fungal growth and drying in their gate-to-grave model, but relied substantially on secondary inventories, proxy materials and assumed distributions of production energy [13]. These studies therefore identify likely environmental hotspots but do not yet provide complete, directly metered industrial inventories for all energy-intensive processing stages.

A more recent laboratory-scale assessment by Volk et al. reported a climate-change impact of 0.3668 kg CO_2_ eq kg^−1^ for an MBC produced in Germany and identified electricity consumption and hemp cultivation as major contributors, although end-of-life treatment was outside the system boundary [90]. Future assessments should separately metre substrate preparation, sterilisation or pasteurisation, incubation-room heating and ventilation, humidity control, pressing, drying and biological inactivation. They should also adopt functional units based on verified packaging performance, such as the quantity of material required to protect a specified product throughout a defined distribution cycle, rather than comparing materials solely on a mass basis.

Protective packaging currently represents the most technologically mature and commercially established application sector for MBCs [15,85]. However, market development remains regionally imbalanced. North America dominates the current industrial landscape owing to early technological development and intellectual property accumulation. Patent analyses indicate that approximately two-thirds of global fungal-material patents are concentrated among a small number of pioneering companies, with Ecovative Design alone accounting for nearly half of all patents related to mycelium packaging technologies [44].

In contrast, the Asia-Pacific region, although relatively late in industrial development, possesses strong long-term growth potential due to abundant agricultural residues, expanding sustainable packaging regulations, and increasing manufacturing demand [10]. Therefore, global industrial development is characterised by a combination of technological concentration in North America and rapid market expansion in emerging economies.

### 5.2. Commercial Packaging Applications

Protective packaging remains the primary commercial application of MBCs. Through near-net-shape biofabrication, mycelium materials can be moulded into highly customised geometries suitable for electronic products, fragile consumer goods, and glass packaging systems [15,85]. Their porous structures provide cushioning, vibration-damping and energy-absorption potential, although comparisons with conventional foams remain dependent on material density, packaging geometry and testing conditions [12,15].

Different application sectors impose distinct functional requirements on MBCs, including mechanical protection, biosafety, thermal insulation, tactile aesthetics, and environmental compatibility. Representative application areas and their associated performance requirements are summarised in Table 7.

Several multinational companies have already incorporated mycelium packaging into commercial product systems. Dell has utilised fungal-derived cushioning materials for electronic packaging, while companies such as LUSH and IKEA have explored mycelium-based inserts and protective components for cosmetics and consumer products [68]. In the food-packaging sector, mycelium-derived films have been investigated as bio-based and biodegradable alternatives to conventional plastic films; however, their suitability for direct food contact remains conditional on validated fungal inactivation, migration, allergenicity and regulatory testing [8,10,84,91].

Beyond packaging, fungal biomaterials are increasingly being investigated for applications in construction, furniture, aerospace systems, and wearable bioelectronics [50,93,94]. These developments demonstrate that fungal-derived biomaterials are gradually transitioning from single-function materials towards multifunctional engineered biomaterial platforms.

### 5.3. Commercial, Regulatory and Sustainability Drivers

The commercialisation of MBC packaging is being encouraged by restrictions on selected single-use plastics, corporate environmental, social and governance commitments, and growing demand for packaging derived from renewable resources [8,9,10]. Nevertheless, policy eligibility and credible environmental claims depend on demonstrated food-contact safety, controlled biological inactivation, recognised compostability testing and life cycle evidence; they cannot be inferred solely from the bio-based origin of the material [10,12,84].

The use of locally available agricultural residues may shorten supply chains, reduce transportation requirements and support distributed production. However, regional differences in feedstock composition, seasonal availability, moisture content and contamination risk can also increase process variability and complicate quality assurance [9,16,68]. Regulatory and market drivers therefore create commercial opportunities, but widespread adoption ultimately depends on reproducible performance, competitive cost, traceability and access to appropriate end-of-life infrastructure.

### 5.4. Cost, Batch Variability, Standardisation and Scale-Up Barriers

Batch-to-batch variability must be distinguished from variation among replicate specimens produced within a single experimental condition. The coefficient of variation may be expressed as CV = SD/mean × 100%. Selected laboratory studies have reported within-condition replicate CV values below 2.5% for some measured properties [95], while an optimised reinforced composite group in another study showed a CV of approximately 11% [80]. However, these values were not obtained from multiple independent industrial batches. A defensible typical batch-to-batch CV for commercial MBC production therefore remains unavailable, and future studies should report results from at least three independently inoculated and manufactured lots rather than only multiple specimens cut from the same panel.

The principal sources of production variability include substrate chemical composition and particle-size distribution, initial moisture content and water activity, spawn viability and loading, fungal strain, mixing uniformity, mould filling mass, compaction density, oxygen and temperature gradients, contamination, incubation endpoint, drying conditions and specimen conditioning. Industrial quality control should therefore combine incoming-substrate specifications, sieving and moisture adjustment, verified spawn viability, automated dosing and mixing, controlled mould filling, and continuous monitoring of temperature, relative humidity, airflow, O_2_ and CO_2_. Thermal imaging, machine vision or near-infrared sensing may support non-destructive detection of incomplete colonisation, moisture gradients and contamination. Final-lot release criteria should include density, dimensions, residual moisture, visible colonisation, contamination status and application-specific mechanical performance, supported by lot traceability and statistical process control [14,16,20,21,55,68].

The cost structure of MBC packaging differs substantially from that of mature EPS manufacturing. Major MBC cost drivers include feedstock preprocessing, sterilisation or pasteurisation, fungal spawn, moulds and trays, controlled incubation space, climate regulation and aeration, manual handling, drying or thermal inactivation, pressing or coating, quality control and the rejection of contaminated or incompletely colonised parts [14,33,55,68]. By contrast, EPS production benefits from short processing cycles, highly automated moulding lines and established high-volume supply chains, although its costs remain sensitive to resin price, steam and energy consumption, tooling and production scale.

A universal peer-reviewed market price per kilogram or cubic metre cannot presently be assigned to MBC packaging because published cost models are product- and process-specific. Moreover, mass-based comparisons can be misleading. In the television-packaging case analysed by Zoungrana et al., the mycelium-based alternatives required approximately 2.5–7.5 times the mass of EPS to occupy the same protective volume [12]. Because material cost per unit volume can be expressed as C_v_ = p_m_ × ρ, where p_m_ is the price per unit mass and ρ is density, an MBC priced identically to EPS on a mass basis would still have an estimated material cost per packaging volume approximately 2.5–7.5 times higher in that particular case. This is a density-based inference rather than a measured market price and does not include possible differences in processing cost, waste generation, protective performance or end-of-life value. Future economic studies should therefore report cost per functional package or per verified protective capacity, together with throughput, labour, energy use, yield and rejection rate.

Near-term scale-up is most likely to benefit from automated substrate dosing and mixing, inoculum dispensing, mould filling and compaction, conveyor-based transport, robotic demoulding, and automated drying and dimensional inspection. Closed-loop control of temperature, humidity, airflow, O_2_ and CO_2_ could reduce spatial heterogeneity during colonisation, while machine vision, thermal imaging and data-driven models could support endpoint determination and early contamination detection [14,20,55,68]. Continuous or cyclic multilayer packed-bed SSF systems may reduce equipment downtime and improve reactor utilisation, but heat accumulation, gas-transfer gradients, contamination control and residence-time distribution remain important constraints [96]. SmF in stirred-tank or airlift bioreactors may be more readily automated for pure-mycelium biomass production, although dewatering, shaping and drying introduce additional downstream operations and energy demands [65]. Consequently, neither continuous SSF nor SmF should be presented as a universally superior route; their suitability depends on whether the target product is a substrate-reinforced packaging component or a purified mycelium material.

No dedicated international consensus standard currently covers the complete production and qualification of MBC packaging. An interim tiered framework is therefore required. Process-level documentation should specify fungal strain, substrate source and particle size, moisture content, inoculum ratio, incubation conditions, biological inactivation procedure and final conditioning. Material characterisation may use ASTM D1621 for compressive properties and ASTM D1622 for apparent density [97,98], while packaging systems may be evaluated under ASTM D4169 distribution-cycle testing [99]. End-of-life claims should be verified using recognised methods such as ISO 14855-1 for aerobic biodegradation under controlled composting conditions and ISO 17088 for compostability specifications [100,101]. These methods were not developed specifically for MBCs, but they provide a more reproducible basis for cross-study and commercial comparison until material-specific standards are established.

Pure-mycelium materials and lignocellulosic MBCs also present different commercialisation pathways. Pure-mycelium systems can provide comparatively homogeneous films, mats and surface structures that are attractive for textile-like, leather-like or other higher-value applications; however, they may require refined nutrient media, controlled surface or submerged cultivation, biomass separation, dewatering, casting and energy-intensive drying [14,40,51,54]. In lignocellulosic MBCs, the agricultural residue functions simultaneously as a nutrient source and structural reinforcement, enabling near-net-shape production and making this route more immediately suitable for compressive cushioning and protective packaging. Its main limitations are feedstock heterogeneity, moisture sensitivity, contamination risk and batch variability [14,55,68]. Accordingly, substrate-reinforced MBCs appear more practical for near-term high-volume protective packaging, whereas pure-mycelium materials may be better suited to higher-value film, coating and textile-related markets.

## 6. Conclusions, Current Limitations and Future Perspectives

### 6.1. Conclusions

This review demonstrates that the performance of mycelium-based composites is not an intrinsic consequence of fungal growth alone, but an emergent outcome of interactions among fungal strain, lignocellulosic substrate, cultivation conditions, material architecture and post-processing. Fungal morphology, substrate particle size and chemistry, moisture content, spawn loading, oxygen transfer, incubation endpoint, pressing and drying collectively regulate density, hyphal connectivity, interfacial bonding and pore structure. These process–structure relationships subsequently determine compressive behaviour, cushioning capacity, thermal insulation, moisture sensitivity and biodegradation. MBCs should therefore be designed as process-dependent engineered materials rather than treated as a single material category with universal properties.

For protective packaging, lignocellulosic MBCs produced predominantly through SSF currently offer the most direct route to near-net-shape cushioning components because the substrate functions simultaneously as a nutrient source and structural reinforcement. SmF is more compatible with the controlled production of pure-mycelium biomass, films and mats, but requires additional dewatering, shaping and drying and should not be regarded as a direct replacement for SSF in all packaging applications. Pressing, fibre reinforcement, polymer modification and surface coating can improve mechanical strength, dimensional stability and barrier performance; however, these gains may be accompanied by increased density, processing energy, cost or reduced end-of-life compatibility. Consequently, the wide property ranges reported in the literature remain strongly dependent on strain, substrate, processing history, specimen geometry, conditioning and testing method.

Current evidence supports MBCs as promising application-specific alternatives to selected petroleum-derived foams, rather than as universally equivalent substitutes for EPS or polyurethane. Available LCAs indicate potential environmental advantages under defined production and end-of-life scenarios, but these advantages vary with material density, electricity source, sterilisation or pasteurisation requirements, incubation control, drying energy, transportation and system boundary assumptions [12,13,90]. Commercial adoption is therefore most realistic where renewable local feedstocks, near-net-shape manufacturing, verified cushioning performance and suitable composting or biological treatment infrastructure can be combined. Reproducible production, functional packaging validation, cost transparency, biosafety and standardised environmental claims remain prerequisites for wider industrial implementation.

### 6.2. Current Limitations and Outstanding Challenges

A fundamental limitation of the current field is its dependence on a relatively narrow range of readily cultivated basidiomycete fungi. Most published studies focus on a small number of species from genera such as *Ganoderma*, *Pleurotus* and *Trametes*, while the material-forming potential of many other saprotrophic, brown-rot, extremotolerant and filamentous fungal groups remains insufficiently investigated [102,103,104]. Even for commonly used strains, the relationships among fungal metabolism, extracellular-matrix secretion, hyphal branching, substrate degradation and interfacial load transfer remain incompletely understood [105,106]. This limits the development of predictive strain–substrate selection rules and means that many fabrication protocols still depend on empirical optimisation.

A second limitation is the poor comparability of published property data. Reported mechanical and physical ranges are affected by fungal strain, substrate composition, particle size, cultivation time, moisture content, pressing conditions, final density, specimen geometry, conditioning and testing protocol [40,55,80]. Many studies report multiple specimens obtained from the same cultivated panel but do not include independently manufactured batches; consequently, within-condition variation cannot be interpreted as true batch-to-batch variability. Although selected laboratory measurements have shown replicate CV values below 2.5% or approximately 11% under specific conditions [80,95], a representative industrial batch CV has not yet been established. The absence of harmonised reporting requirements, reference materials and MBC-specific performance standards further restricts quantitative comparison, meta-analysis and industrial quality assurance [21].

Service-life requirements also remain difficult to reconcile with biological end-of-life objectives. MBCs are generally sensitive to moisture uptake, humidity cycling and biological degradation, while reinforcement, hydrophobic coatings and polymer impregnation can alter compostability, disintegration rate and ecotoxicological performance [18,36,37,38,39,83]. Improvements in water resistance or durability should therefore not be interpreted as environmentally neutral unless coating composition, migration, biodegradation and composting behaviour are evaluated together. Thermal inactivation can prevent continued fungal growth and sporulation, but it does not by itself demonstrate the absence of pre-formed metabolites, allergenic proteins, substrate-derived contaminants or microbial regrowth under humid conditions [82,84]. These limitations are especially important for food-contact, indoor and consumer-facing applications.

Industrial translation remains constrained by long cultivation cycles, sensitivity to contamination and environmental gradients, limited process automation, drying or inactivation energy, variable feedstock quality and the rejection of incompletely colonised components [14,16,33,65,68]. Published economic models are process- and product-specific and do not yet provide a transferable commercial cost per kilogram, unit volume or functional package. Similarly, existing LCAs differ in functional units, density assumptions, electricity mixes and end-of-life scenarios, and some rely on aggregate or secondary energy inventories rather than direct industrial measurements of pasteurisation, incubation control and drying [12,13,90]. Commercial cost, throughput, yield, rejection rate and directly metered environmental inventories therefore remain important evidence gaps.

This review is also subject to limitations arising from the available evidence. Because the underlying studies employ heterogeneous materials, processing routes, test conditions and functional units, the reported performance gains could not be pooled into a formal quantitative meta-analysis. Commercial-scale cost, production yield and quality-control data are frequently proprietary or incompletely reported, while emerging topics such as fungal bioelectronics and living mycelium systems remain supported primarily by proof-of-concept studies. The conclusions of this review should therefore be interpreted as a critical synthesis of current evidence rather than as universal performance specifications or commercially validated design rules.

### 6.3. Future Research and Industrialisation Priorities

Future biological research should move beyond repeated evaluation of a small number of established species and establish application-oriented fungal screening programmes. Additional saprotrophic basidiomycetes, including brown-rot taxa, may provide different colonisation rates and lignin-rich residual substrate structures, although excessive polysaccharide degradation could also weaken the reinforcement phase and must be evaluated experimentally [102,103]. Extremotolerant fungi may offer useful traits for manufacturing under fluctuating temperature, moisture or osmotic conditions [104], whereas rapidly growing filamentous ascomycetes could potentially shorten production cycles but would require particularly rigorous screening for secondary metabolites, allergens and pathogenicity [84,107]. Mycorrhizal fungi are scientifically relevant to fungal–plant interface research but should be treated as longer-term candidates because many depend strongly on living plant partners and are difficult to cultivate in independent, high-throughput manufacturing systems [108]. Screening should therefore assess growth rate, substrate compatibility, hyphal architecture, bonding efficiency, metabolite profile, sporulation and biosafety rather than selecting strains on colonisation speed alone.

Manufacturing research should prioritise reproducibility before increasing production volume. Automated substrate dosing, moisture adjustment, inoculum dispensing, mixing, mould filling, compaction, robotic demoulding and controlled drying should be integrated with closed-loop monitoring of temperature, relative humidity, airflow, O_2_ and CO_2_ [14,55,65]. Machine vision, thermal imaging and data-driven prediction may support early contamination detection and objective incubation-endpoint determination [20]. Continuous or cyclic packed-bed SSF systems could improve reactor utilisation, but heat accumulation, gas-transfer gradients, contamination control and residence-time distribution require further validation [96]. Distributed manufacturing based on regional agricultural residues may reduce transport and feedstock costs, but it should be implemented only with incoming-substrate specifications, preprocessing protocols and lot traceability capable of controlling seasonal and geographical variability [9,16,68].

Future material development should evaluate reinforcement and coating strategies through multi-criteria optimisation rather than maximising a single mechanical or barrier property. Natural fibres can improve load transfer but may also introduce hydrophilicity, poor dispersion, variable interfacial bonding and degradation during cultivation [73]. Nanocellulose, chitin nanofibres and bio-based polymers may enhance strength and moisture resistance, although their benefits must be weighed against material cost, processing complexity, dispersion requirements and changes in density [19,43,68]. Surface treatments should be evaluated simultaneously for water-vapour and liquid-water resistance, food-contact migration, composting rate, ecotoxicity and coating detachment [36,37,38,81]. The preferred strategy should provide sufficient protection during the intended service period while still allowing predictable biological treatment at end of life.

Industrial qualification requires a harmonised minimum reporting framework covering fungal identity, substrate source and composition, particle-size distribution, moisture content, inoculum ratio, cultivation conditions, pressing, drying, density, specimen conditioning and test geometry. Mechanical and packaging validation may initially draw on ASTM D1621, ASTM D1622 and ASTM D4169, while biodegradation and compostability claims should be assessed using recognised methods such as ISO 14855-1 and ISO 17088 [97,98,99,100,101]. Studies should report independent production lots, CV values, yield and rejection rate and should provide open process–property datasets suitable for statistical modelling and external validation [20,21]. Future LCA and techno-economic assessments should use functional packaging units and directly metre sterilisation or pasteurisation, environmental control, pressing, drying, inactivation, transportation and end-of-life treatment [12,13,90].

Living mycelium materials may enable sensing, electrical response, adaptive behaviour and limited self-repair [109,110,111,112], but biological activity should be retained only when it provides a necessary and verifiable function. Risk-by-design assessment should include strain identity, pathogenicity, mycotoxin potential, allergenicity, spore and hyphal-fragment release, genetic stability across repeated growth cycles, mutation, microbial contamination and behaviour under foreseeable temperature and humidity conditions [84]. Possible controls include non-sporulating or well-characterised strains, physical encapsulation, restricted nutrient availability, defined operational lifetimes, environmental monitoring and validated post-use inactivation. Passive cushioning and food-contact packaging should generally use fully inactivated materials unless continued viability provides a demonstrated benefit and has passed application-specific regulatory and biosafety assessments. Accordingly, near-term industrial priorities should focus on reproducible passive MBC packaging, standardised testing, cost reduction and metered environmental performance; engineered living systems should remain a longer-term direction pursued alongside explicit containment and safety frameworks.

## Figures and Tables

**Table 1:** Comparison of representative physical and mechanical properties between mycelium-based composites (MBCs) and expanded polystyrene (EPS).

Performance Indicators	EPS	MBCs	Representative Literature
Compressive Strength (MPa)	0.10–0.35 (Packaging-grade EPS)	0.05–0.50 (in its natural state)	[34,35]
		0.80–2.00+ (after hot pressing)	
Thermal Conductivity (W/m·K)	0.030–0.040	0.040–0.080	[31]
Young’s Modulus (MPa)	2.50–5.00	1.00–15.00 (strongly dependent on density and processing conditions)	[30,35]
Density, kg/m^3^	15.0–50.0	50.0–300.0	[34]
End-of-life	Persistent and non-biodegradable; recovery depends on collection, sorting and recycling infrastructure	Potentially biodegradable under controlled conditions; degradation rate and completeness depend on density, heat treatment, coating formulation, microbial activity, temperature, moisture and disposal environment	[4,5,6,7,36,37,38,39]

The values are indicative study-specific ranges rather than universal material constants. Direct comparisons depend on EPS grade, MBC fungal strain, substrate composition, density, pressing conditions, specimen geometry, conditioning and testing protocol. Biodegradation claims apply only to specified test environments and should not be inferred from short-term mass loss or physical disintegration alone.

**Table 2:** Representative reported biochemical characteristics and mechanical property ranges of fungal species used in MBC fabrication.

Fungal Species	Reported Cell-Wall Tendency	Young’s Modulus (MPa)	Strength (MPa)	Performance Characteristics	Representative Literature
*Ganoderma lucidum*	Relatively high chitin content	45.0–70.0	1.0–2.0	High rigidity, high modulus, brittle	[11,40]
*Pleurotus ostreatus*	Moderate chitin content	10.0–30.0	0.5–1.0	High flexibility, high strain capacity	[1,11]
*Trametes versicolor*	Moderate chitin content	20.0–40.0	0.6–1.2	Moderate performance	[1]

The values shown are study-specific reported ranges and should not be interpreted as intrinsic constants of individual fungal species. Their upper and lower bounds vary with fungal strain or isolate, substrate chemistry and particle size, moisture content, cultivation time, composite density, pressing and post-treatment conditions, specimen geometry, and testing protocol. Cross-species comparisons are therefore indicative rather than directly quantitative.

**Table 3:** Representative effects of substrate selection and preparation parameters on the structure and mechanical performance of MBCs.

Research Content	Experimental Methods	Key Findings	Reference
Effect of particle size and fibre orientation on compressive strength	Three single-grade and mixed-grade mixtures	Particle size and orientation significantly affect ultimate strength (*p* < 0.05)	[60]
Effect of substrate digestibility on chitin content	Microcrystalline cellulose vs. glucose	Indigestible substrates induce an increase in chitin synthesis of ~80%	[25]
Combined effects of fungal strain, substrate formulation, particle-size distribution, and inoculum level	Sequential screening of 10 fungal strains, 17 substrate formulations, particle-size distributions, and inoculum levels	*Ganoderma resinaceum* combined with reed straw/corn cob, a particle-size distribution of 0.075–2.000 mm, and 7.5% inoculum produced 190.99 kPa compressive strength at 10% deformation, approximately 1.9 times that of 18 kg m^−3^ EPS.	[61]
Combined effects of inoculum level, substrate particle size, and water addition	Three-factor, three-level orthogonal experimental design	An inoculum level of 10%, a particle size of 10 mm, and water addition of 60% produced a bending strength of 417.43 kPa; cushioning performance remained below that of EPS	[62]
Effect of micro-particle addition on density and modulus	Addition of <100 µm particles	Density + 30%, compressive elastic modulus + 156%	[63]
Effect of substrate type on density	Sawdust, maize husks, rice straw	Sawdust has the highest density (0.48 g/cm^3^), with a positive correlation between particle size, density and performance	[58]
Effect of particle size distribution on porosity and strength	Narrow distribution vs. broad distribution	A broad distribution reduces porosity by 22% and increases strength by 34%	[56]

The reported values were obtained using different fungal species, substrates, densities, cultivation conditions, specimen geometries, and mechanical test protocols. They should therefore be interpreted as study-specific process–property relationships rather than directly comparable material rankings.

**Table 4:** Comparison between solid-state fermentation (SSF) and submerged fermentation (SmF) systems for mycelium-based material fabrication.

Comparison Criteria	SSF	SmF
Main Product	Mycelium-based composites (MBCs)	Pure mycelium membranes and nanopapers
Typical Structures	Foam-like, block-shaped cushioning pads	Film, sheet-like
Dominant Performance Profile	Bulk compressive and cushioning performance governed by the lignocellulosic substrate network, density and colonisation uniformity	Comparatively uniform films and mats with emphasis on tensile flexibility, surface quality and continuity
Process characteristics	Static cultivation in moulds with direct colonisation of lignocellulosic residues	Sterile, agitated bioreactor cultivation followed by biomass recovery and shaping
Cost level	Generally lower, but affected by sterilisation, environmental control, contamination prevention, and drying	Generally higher because of bioreactor operation, aeration/agitation, sterility control, and downstream biomass recovery
Packaging application positioning	Cushioning and protective packaging	Films, coatings, and speciality sheet products
Primary scale-up constraints	Non-uniform heat and mass transfer, contamination, long cultivation cycles, and batch variability	Bioreactor capital cost, aeration and agitation energy, sterility control, and downstream processing
Principal Downstream Trade-Off	Requires mould handling, complete colonisation, drying and biological inactivation; substrate heterogeneity can increase batch variability	Requires biomass separation, dewatering, shaping or casting and drying; downstream recovery may increase energy and cost

This comparison is qualitative. Performance, cost and scale-up potential depend on fungal strain, substrate or nutrient medium, reactor configuration, residence time, energy consumption, downstream recovery and target product [40,51,54,65,66].

**Table 5:** Structure–property relationships of key microstructural components in mycelium-based composites.

Structural Element	Form	Impact on Performance
Mycelial network	Three-dimensional branching, physical entanglement	Provides tensile strength and overall stability
Air gaps/Pores	Intercellular voids, micron-scale channels	Enhance thermal insulation and sound absorption, reduce density, but reduce strength
Substrate particles	Skeletal support, lignocellulose	Provide compressive modulus, influence mycelial growth rate
Interface layer	Biochemical bonding, mechanical interlocking	Determines the material’s fracture energy and fatigue resistance

**Table 6:** Qualitative comparison of reinforcement and surface-modification strategies for mycelium-based composites.

Strategy and Key References	Primary Performance Contribution	Principal Limitations	Relative Cost and Scale-Up Implications	End-of-Life Considerations
Densification and hot pressing [40,78,79]	Increases density, stiffness, compressive strength and dimensional stability	Increased mass; reduced porosity, cushioning and thermal insulation; possible brittle behaviour at excessive densification	Low-to-moderate process complexity; compatible with batch pressing, but adds energy demand and processing time	Introduces no foreign reinforcement, but higher density and thermal treatment may slow disintegration
	Increased mass; reduced porosity, cushioning and thermal insulation; possible brittle behaviour at excessive			
Natural-fibre reinforcement [17,80]	Provides additional load-transfer pathways and may improve strength and toughness	Hydrophilicity, swelling, poor dispersion, variable interfacial bonding and possible fibre degradation during cultivation	Relatively low material cost when local fibres are used; pretreatment, alignment and controlled mixing add complexity	Predominantly bio-based, but chemical pretreatment and increased moisture uptake may affect degradation behaviour
Carbon-nanotube reinforcement [18,68]	May improve electrical conductivity, sensing capability and mechanical reinforcement	Dispersion difficulty, occupational exposure, high material cost and dependence of self-repair claims on continued biological activity	High material and processing burden; currently unsuitable for high-volume disposable packaging	Recovery, ecotoxicity and compatibility with composting remain uncertain
Cellulose nanocrystals and chitin nanofibres [19,68]	Provide nanoscale interfacial reinforcement and may improve stress transfer and barrier performance	Hydrophilicity, agglomeration and sensitivity to dispersion quality	High isolation, purification and dispersion requirements compared with conventional natural fibres	Renewable origin does not guarantee low impact; processing energy and composting behaviour require verification
Hybrid bio-based polymer systems [42,68]	May improve water resistance, tensile performance, flexibility and dimensional stability	Additional mass, possible interfacial incompatibility and formation of relatively continuous polymer phases	Moderate-to-high material and fabrication complexity; better suited to higher-value products	Biodegradability depends on polymer identity, loading, continuity and the final composite formulation
Natural wax, oil or resin surface treatments [36,37,38]	Improve liquid-water resistance and reduce short-term water uptake	Coating non-uniformity, abrasion, cracking and limited control of long-term water-vapour transmission	Low-to-moderate complexity and potentially compatible with existing coating processes	May delay composting or introduce migration and ecotoxicological concerns; coated products require separate end-of-life testing

The relative cost and scale-up assessments are qualitative and refer to additional processing burden rather than universal market prices. Performance gains cannot be directly pooled because the underlying studies use different material systems and testing conditions.

**Table 7:** Key performance requirements and corresponding advantages of mycelium materials across different application areas.

Application Area	Core Performance Requirements	Advantages of Mycelium Materials	Key References
Electronic products	High dynamic damping, dimensional accuracy	Cushioning and energy-absorption potential, together with near-net-shape forming capability	[12,15]
Food and beverage	Biosafety, hygiene, moisture resistance and food-contact compliance	Potential bio-based and compostable formats, subject to validated fungal inactivation, migration and food-contact safety testing	[10,84,91]
Beauty and personal care	Tactile aesthetics, brand storytelling	Natural velvet texture, three-dimensional malleability	[68]
Furniture and architecture	Structural strength, thermal insulation, sound insulation	Mechanical properties similar to cork, low thermal conductivity	[50,92]
Aerospace and cutting-edge applications	Radiation resistance, stability in extreme environments	Dense mycelial network protection, potential for self-repair	[93]

## Data Availability

No new data were created or generated in this review article. All information and data discussed in this study were obtained from previously published literature, which are publicly available and appropriately cited in the reference list. The datasets and information summarized in this review were compiled and analysed from published sources.
